# Psychometric properties of the Infant Motor Profile (IMP): A scoping review protocol

**DOI:** 10.1371/journal.pone.0277755

**Published:** 2022-11-16

**Authors:** Luiza Ribeiro Machado, Carolina Fioroni Ribeiro da Silva, Mijna Hadders-Algra, Eloisa Tudella

**Affiliations:** 1 Physical Therapy Department, Federal University of São Carlos, São Carlos, São Paulo, Brazil; 2 Department of Paediatrics, Institute of Developmental Neurology, University of Groningen, University of Medical Center Groningen, Groningen, The Netherlands; Duta Wacana Christian University School of Medicine / Bethesda Hospital, INDONESIA

## Abstract

**Introduction:**

The IMP is a novel video-based instrument to assess motor behavior of infants. It evaluates gross and fine motor behavior in five domains: variation, adaptability, symmetry, fluency, and performance. The latter assesses motor milestones, the other four domains assess qualitative aspects of movements. Literature suggests that it is a promising tool for pediatric health care, as its assists early detection of neurodevelopmental disorders and facilitates the design and monitoring of early intervention. This, this scoping review (ScR) aims to evaluate the psychometric properties of the Infant Motor Profile (IMP).

**Material and methods:**

A systematic search will be conducted to identify relevant studies up to October 15, 2022. All papers published in English that evaluated or used the IMP in children under two years of age will be included. The search will be performed in Pubmed, Lilacs, PEDro, Scielo, CINAHL, Embase, Web of Science, Ovid PsycINFO, Cochrane Database of Systematic Reviews, as well as in gray literature sources following the University of Toronto library guidelines. Standardized data extraction forms (Excel Tables) will be used to collect information. The Preferred Reporting Items for Systematic Reviews and Meta-Analyses extension for the Scoping Reviews (PRISMA-ScR) Checklist and JBI guidelines will be taken into consideration for results analysis and reporting.

**Discussion:**

This Scoping Review will summarize available knowledge on the psychometric properties of the IMP. By proving that IMP is a reliable tool, a valid predictor of neurodevelopmental outcomes and a responsive instrument to measure change induced by early intervention, this will facilitate the implementation of the IMP in pediatric health care. It will assist the detection of infants at high risk of neurodevelopmental disorders, and it will facilitate the design of the tailor-made early intervention.

**Scoping review protocol registration:**

This scoping review protocol has been registered at Open Science Framework (OSF) (https://doi.org/10.17605/OSF.IO/4HYKZ).

## Introduction

Infants who spent the beginning of extrauterine life in a neonatal intensive care unit are at high risk of neurodevelopmental disorders (NDD). It has been estimated that about 25% of these infants are diagnosed with NDD [1]. A major challenge for clinicians is to detect the infants who will be diagnosed with NDD at an early age. The challenge occurs because of the dramatic developmental changes that occur in the brain during the first two postnatal years [2]. Meanwhile, this period with high neuroplasticity offers the best opportunities for successful early intervention [3].

It has been established that the best tools for early detection consist of early magnetic resonance imaging and general movement assessment [3]. However, magnetic resonance imaging facilities are not everywhere available and general movement assessment can only be performed until the corrected age of five months. Other instruments for the prediction of NDD, that are applicable in older infants, are the Hammersmith Infant Neurological Examination [4], and the Standardized Infant NeuroDevelopmental Assessment [5]. These instruments are however less appropriate to monitor the infant’s motor development and the effect of early intervention. Other instruments that may be used to monitor the infant’s motor progress are the Test of Infant Motor Performance [6] and the Alberta Infant Motor Scale [7]. Yet, these methods have only a limited capacity to predict NDD. They also mainly evaluate gross motor functions. The novel instrument Infant Motor Profile (IMP) [8] is the only instrument that evaluates gross and fine motor development and combines predictive and responsive evaluative properties for NDD [9].

The IMP is based on the Neuronal Group Selection Theory. According to Neuronal Group Selection Theory, human motor development is characterized by two phases, i.e., the phases of primary and secondary variability [10,11]. During the first phase, individuals present a wide variety of movements. The afferent feedback of these movements is used for further sculpting of the brain, but cannot be used to adapt movements to the specifics of the situation. In the phase of secondary variability, sensory information associated with own exploratory, trial and error activities can be used to learn to select the most efficient movement strategy in each situation. This means that during the phase of secondary variability, motor behavior is characterized by a varied repertoire and the development of adaptive movements [12]. In contrast, infants with an early lesion of the brain may present with a reduced movement repertoire being expressed by reduced variation and the presence of stereotyped movements. Additionally, an early lesion of the brain may result in impaired adaptability due to a) the absence of specific optimal strategies (due to repertoire reduction); b) a reduced exploratory drive, and c) impaired processing of sensory information, which interferes with the process of learning to select adaptive strategies [13].

The IMP is a video-based instrument for infants aged three to 18 months, or rather to the moment when the child is able to walk independently for some months. It is based on the infant’s self-generated movements during a playful interaction with the assessor. The IMP consists of 80 items that provide information on the infant’s motor behavior in five domains: variation, adaptability, symmetry, fluency and performance. The domains of variation and adaptability are based on the Neuronal Group Selection Theory. The three other domains are traditional domains of motor development. This also means that the IMP has four domains evaluating the quality of motor behavior and one domain that evaluates performance, i.e., motor skills or motor milestones. The IMP results in five domain scores and one total score. The assessment itself takes about 15 minutes and its off-line scoring about 10 minutes. Recently, the IMP-manual became available, including many video examples, an app to calculate the scores and IMP-norms (percentile values) [14]. Additionally, the IMP does not require a specific tool kit, but toys that can be easily acquired [8,15–17].

The aim of this scoping review is to evaluate the psychometric properties of the IMP, including its predictive validity. Here it is important to realize that prediction at early age of developmental outcome in later life is never perfect, as the young nervous system develops very rapidly. Due to the rapid changes, infants may outgrow their initial impairments, however, they also may grow into a deficit [9]. This also means that it is recommended not to rely on a single clinical tool, but to also use complementary clinical tools [18]. The Joanna Briggs Institute (JBI) Database of Systematic Reviews and Implementation Reports, and Cochrane Database of Systematic Review indicate that reviews on the psychometric properties of the IMP are lacking. Therefore, we aim to offer a systematic overview of the properties of IMP, allowing health professionals and researchers to decide whether the IMP suits their goals of assessing infant motor behavior.

## Review question(s)

To review the literature on the psychometric properties of the IMP. Specific questions addressed are:

- How reliable is the IMP, in particular, which values have been reported for the intra- rater and the inter-rater agreement?
- Do IMP domains reflect the neuromotor function of infants (construct validity)?
- What are the concurrent validity values of the IMP reported in the literature, and on which comparisons are these validity values-based?
- Is the IMP able to detect forthcoming impairments and disability proving its predictive validity?
- Does the IMP have adequate responsivity to detect the effect of early intervention?

## Material and methods

The proposed scoping review will embrace the methodology for scoping reviews, as stated by JBI [19]. The scoping review will use the Preferred Reporting Items for Systematic Reviews and Meta-Analyses extension for the Scoping Reviews (PRISMA-ScR) checklist (S1 File) for the writing of this study report [20]. This scoping review protocol was registered in the Open Science Framework Register (https://doi.org/10.17605/OSF.IO/4HYKZ).

## Inclusion criteria according to PCC

### Participants

This scoping review will consider literature that evaluates and/or uses the IMP in children under the corrected age of two years old, of any sex and culture, living in regional, rural, and remote communities.

### Concept

This scoping review will consider literature that explores the psychometric properties of IMP, i.e., its reliability, concurrent, construct and predictive validity and its responsivity.

### Context

This scoping review will include literature published in English. The selected literature may study children under two years old recruited in various settings, such as community, primary care, healthcare centers, and hospitals.

### Types of sources

This scoping review will evaluate published and unpublished primary and secondary studies on this subject, regardless of the rigor to align the review question to the objectives.

### Search strategy

The search strategy aims to find both published and unpublished literature. The JBI’s three-stage search strategy [19] will be applied to conduct this review (full search strategy in Table 1). The first stage involved an initial limited search of PubMed and Science Direct to identify keywords contained in the text and title of relevant literature. These keywords were used with index terms to develop a search strategy for each database and gray literature source. For the second stage, this scoping review used all identified keywords and index terms to tailor the search to each information source. The third stage involved a search of the reference lists of included citations for additional literature. When appropriate, the reviewers will contact the authors for additional information.

**Table 1:** Search strategy.

Search	Query	Records retrieved
#1*	“Infant” AND “Infant Motor Profile” [Mesh] / “Children” AND “Infant Motor Profile” [Mesh] at Pubmed Central: PMC and ScienceDirect (Elsevier)	65
#2*	“Infant” [AND] “Infant Motor Profile” [Mesh] / “Children” AND “Infant Motor Profile” [Mesh] searched in all databases and grey literature	819 (346 in the databases, and 473 in grey literature)
#3*	#1 and # 2	884

# = phase; Mesh = Medical Subject Headings.

*The search was performed up to October 15, 2022, and only considered materials published in English.

This scoping review will accept literature published in English, taking into account the reviewers’ proficiency language level, enabling a good quality of evidence selection and data extraction. It will consider the literature published until October 15 of 2022.

For published studies, the reviewers will conduct electronic searches in the following databases: CINAHL with Full Text, Web of Science–Main Colection (Clarivate Analytics), Lilacs—*Literatura Latino-Americana e do Caribe em Ciências da Saúde*, PEDro—Physiotherapy Evidence Database, Embase (Elsevier), Pubmed Central: PMC, ScienceDirect (Elsevier), PsycINFO (APA), ScIELO.ORG, and Cochrane Library. The search for unpublished studies and gray literature will be conducted based on the University of Toronto Guidelines [21] and will include the sources: Google scholar, WHO ICTRP Search Portal, OpenGrey, OpenDOAR, Childlink, Zetoc, health services, health research websites, open access websites, contact with authors, repositories and catalogs. Finally, the search will use the IMP-manual [14] and its literature references.

### Source of evidence selection

**Fig 1:**
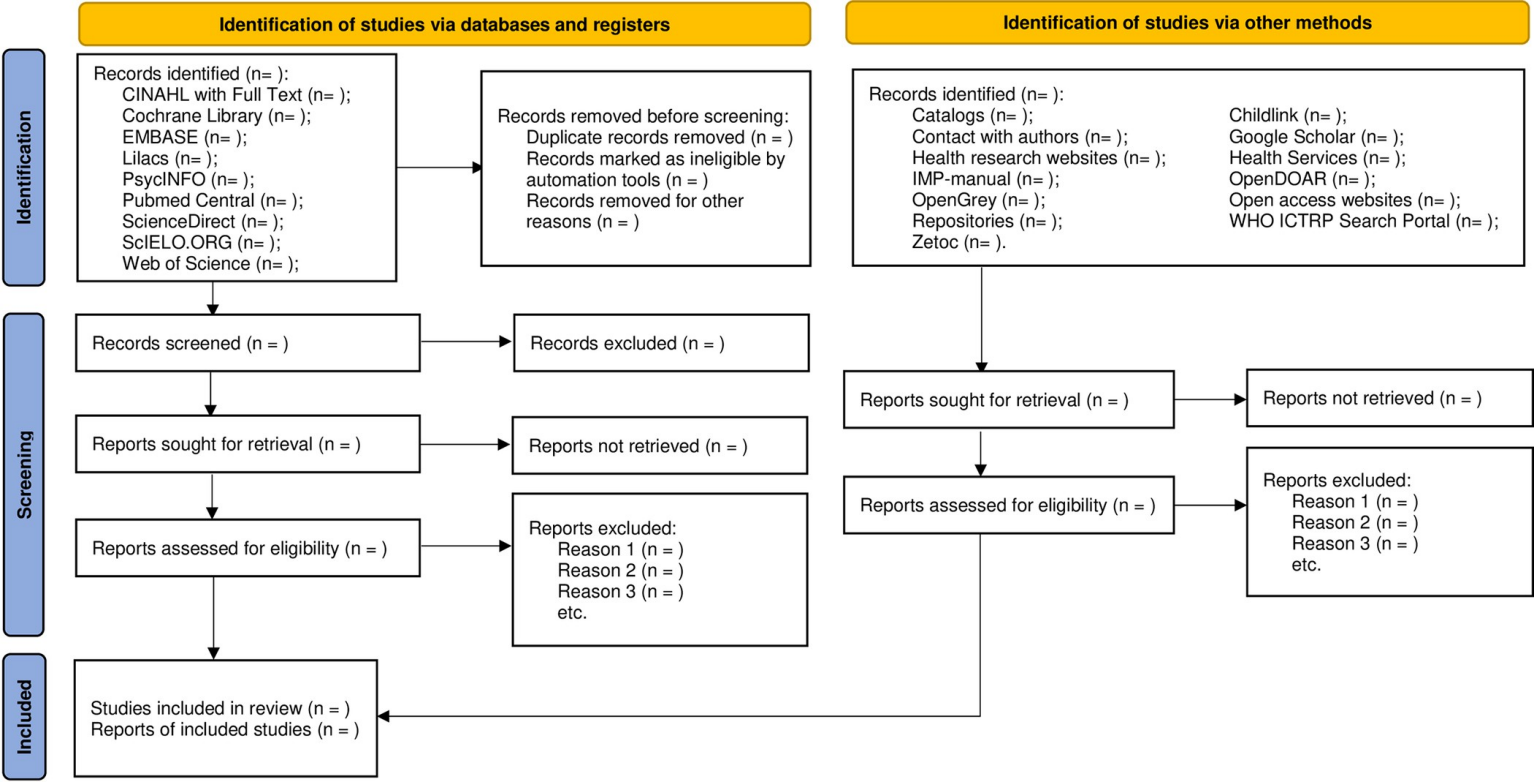
Flow diagram model, which will be used in the scoping review. Flow diagram based on the schematic overview of Flow Diagrams available at PRISMA.

In sequence, this scoping review will compile and upload figures and written literature into the management system EndNote X7 to remove the duplicate literature. Subsequently, two independent reviewers (LM and CS) will screen the titles and abstracts according to the inclusion criteria. In phase one of the project, i.e. the phase during which the search algorithm and literature selection were tested (see Table 1), the two reviewers reached a high level of agreement (86% agreement and kappa = 0.824). Relevant papers will be fully analyzed, considering the inclusion criteria by the reviewers. Full- text papers not included in the scoping review will be specified and reported. In case of any disagreement between the first and second reviewers, the third reviewer (ET) will be consulted for final decision. The results of the search will be reported in full in the final scoping review, and presented in a Preferred Reporting Items for Systematic Reviews and Meta-Analyses extension for the Scoping Reviews (PRISMA-ScR) [20] flow diagram (Fig 1).

### Data extraction

The first and second reviewers will independently perform the general data extraction and management from the selected literature using a prestructured and predefined Excel form. The data extraction form includes information on the authors, journal, and year of publication, the groups studied (size and background), the psychometric properties evaluated and how it was performed, the results on the psychometric properties, and the risk of bias.

The data extraction Excel form is available in Supplemental material II (S2 File).

### Data analysis and presentation

The extracted data will be presented in tabular form in a manner that aligns with the aims of this scoping review report. The evaluation of IMP’s reliability focuses on intra-rater and inter-rater agreements. The evaluation of the various forms of validity will pay attention to the nature of the groups studied, e.g., whether the infants studied are at high or low risk of neurodevelopmental disorders. The assessment of construct validity evaluates which risk factors will be associated with low IMP-scores. Construct validity will be confirmed when low-IMP scores are associated with social, prenatal, perinatal, and neonatal risk factors. The evaluation of the concurrent validity will report the association of IMP-scores with scores on other neurodevelopmental assessments, therewith also paying attention to the type of comparison instruments used. Moderate associations between IMP-scores and scores on other neurodevelopmental assessments will confirm IMP’s concurrent validity. High correlations are not expected, as this would demonstrate that the IMP measures the same as the other instruments. Predictive validity addresses the question of how well low IMP-scores predict the neurodevelopmental outcome at a later age. It is confirmed when low IMP scores predict the neurodevelopmental outcome at a later age. Specific attention will be paid to the age and the type of outcome (e.g., CP, developmental delay, or cognition). Finally, IMP’s responsivity will be determined by evaluating intervention studies that used the IMP as an outcome measure. IMP’s responsivity is confirmed when the IMP is able to measure a difference in the effect of two types of early intervention. A narrative summary will accompany the tabulated results and will describe how the results relate to the reviews’ aims and question/s.

### Data Management Plan

The ideal place for the data of the Scoping Review will be the Open Science Framework (https://osf.io/). The data will be published in spreadsheet format,.xlsx, being unzipped and not encrypted. Double typing will be performed to avoid possible errors in code and typing. The data will be stored on two external hard drives and Google Drive to maintain security and prevent data loss. It will be available for 20 years. The intellectual property of the content remains on the authors and journals that published the papers, as they are properly cited.

## Discussion

This Scoping Review will summarize available knowledge on the psychometric properties of the IMP. By proving that IMP is a reliable tool, a valid predictor of neurodevelopmental outcomes and a responsive instrument to measure change induced by early intervention, this will facilitate the implementation of the IMP in pediatric health care. It will assist the detection of infants at high risk of neurodevelopmental disorders, and it will facilitate the design of the tailor-made early intervention.

### Limitations of the study design

We will only review material published in English. Therefore, we may lose some information available in other languages such as Chinese or German.

## Supporting information

S1 File PRISMA-ScR (Preferred Reporting Items for Systematic Reviews and Meta-Analyses extension for the Scoping Reviews) checklist. (PDF)

S2 File Excel data extraction form. (XLSX)

## Abbreviations

IMP: Infant Motor Profile
ScR: Scoping review
JBI: Joanna Briggs Institute
PRISMA-ScR: Preferred Reporting Items for Systematic Reviews and Meta-Analyses extension for the Scoping Reviews
NDD: Neurodevelopmental disorders
